# SOCR data dashboard: an integrated big data archive mashing medicare, labor, census and econometric information

**DOI:** 10.1186/s40537-015-0018-z

**Published:** 2015-07-17

**Authors:** Syed S Husain, Alexandr Kalinin, Anh Truong, Ivo D Dinov

**Affiliations:** Statistics Online Computational Resource (SOCR), UMSN, 400 N. Ingalls Health Behavior and Biological Sciences Prechter Bipolar Research Fund Michigan Institute for Data Science University of Michigan, Ann Arbor, MI 48109 USA

## Abstract

**Introduction:**

Intuitive formulation of informative and computationally-efficient queries on big and complex datasets present a number of challenges. As data collection is increasingly streamlined and ubiquitous, data exploration, discovery and analytics get considerably harder. Exploratory querying of heterogeneous and multi-source information is both difficult and necessary to advance our knowledge about the world around us.

**Research design:**

We developed a mechanism to integrate dispersed multi-source data and service the mashed information via human and machine interfaces in a secure, scalable manner. This process facilitates the exploration of subtle associations between variables, population strata, or clusters of data elements, which may be opaque to standard independent inspection of the individual sources. This a new platform includes a device agnostic tool (Dashboard webapp, http://socr.umich.edu/HTML5/Dashboard/) for graphical querying, navigating and exploring the multivariate associations in complex heterogeneous datasets.

**Results:**

The paper illustrates this core functionality and serviceoriented infrastructure using healthcare data (e.g., US data from the 2010 Census, Demographic and Economic surveys, Bureau of Labor Statistics, and Center for Medicare Services) as well as Parkinson’s Disease neuroimaging data. Both the back-end data archive and the front-end dashboard interfaces are continuously expanded to include additional data elements and new ways to customize the human and machine interactions.

**Conclusions:**

A client-side data import utility allows for easy and intuitive integration of user-supplied datasets. This completely open-science framework may be used for exploratory analytics, confirmatory analyses, meta-analyses, and education and training purposes in a wide variety of fields.

## Introduction

### State of open-science

Open-science refers to a new paradigm liberalizing access, advancement, control, accreditation and ownership of scientific knowledge, information resources (e.g., data), and decision-making instruments (e.g., software tools). In open-science settings, the entire community has open unrestricted access to resources incentivizing user participation at different levels (e.g., consumption, mashing, development), enabling social interactions where the collective outcome is more than the sum of its individual parts, novice learners and experts can choice, contribute to and debate concepts, algorithms, analytics, results and theoretic principles. This paradigm requires a critical mass of participation, commitment for trust, diversity, sharing and cooperation. Outcome products often take unexpected and innovative turns, looking at problems from different angles and employing expertise, methods and services, which initially may appear as not-interoperable. There are many examples of successful open-science initiatives. Two of these are the Polymath project [1] and the Mozilla Open-Science [2]. Polymath1 problem identified by the Polymath community involved searching for a new combinatorial proof to the Hales–Jewett theorem [3]. The project morphed into multiple independent threads, which led to a solution of the problem within several months, using constructive contributions from dozens of people. An international team of scientists and engineers participated in Mozilla Open-Science 52-h coding marathon to enhance open science lessons, learning materials, teaching tools, and software resources and establish minimal standards for open science education. This virtual activity used open-resources (e.g., GitHub) to establish reproducible research guidelines, develop and enhance teaching materials in a diverse array of scientific disciplines (bioinformatics, medical imaging, oceanography, social science, etc.)

There are now over 1 billion web-sites hosted on millions of Internet servers [4]. This widespread availability of information provides web access to resources that can be efficiently shared with minimal barriers to content and data. Yet, there are significant barriers to effective open-science. Some of the challenges include lack of interoperability or compatibility of resources, licensing restrictions, federal, state and local regulations, mistrust in attribution, ineffective infrastructure, and discipline boundaries.

### Challenges in managing, fusing, processing, servicing and understanding heterogeneous data

Big Data is ubiquitous in many, if not all, scientific disciplines, applied studies and research explorations. Its characteristics include heterogeneity, multiple-scales, incongruent space-time sampling, format complexity, privacy restrictions, and multi-source provenance. There are significant, unique and impeding challenges associated with Big Data modelling, handling, analytics, interpretation and progress of extracting information and gaining knowledge from it. Each step in the complete workflow from data acquisition, to data storage, servicing, archival, manipulation and processing present problems that need to be overcome to enable the course of information extraction and decision-making. Lack of standards, unstructured data formats and aggregation of data (elements and cases) inhibit the semantic content processing, search and holistic data understanding. Data volume is not the only bottleneck. In many applications, the data complexity and heterogeneity frequently constrain the use of established methods, techniques, software tools and services. One of the paramount feature of Big Data is its temporal dynamism. The value of models, inference, and findings presented using a stale data repositories rapidly depreciate with time [5]. Just like the formulation of conditional probability and Bayesian inference [6] advanced our understanding of marginal and joint probabilities [7], data mashing, the process of fusion of information from disparate resources, has a potential to revolutionize our understanding of natural processes and complex phenomena.

### Big data analytic infrastructure

**Fig. 1:**
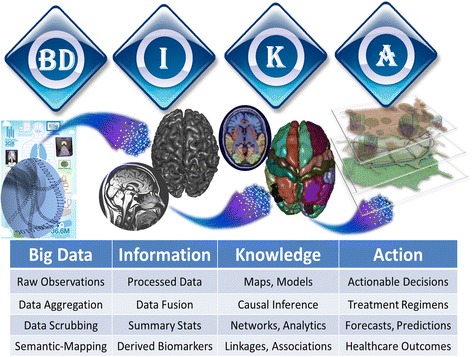
An example of the Big Data Analytics workflow, in the context of clinical neuroscience. This schematic illustrates the Big Data analytics protocol describing the transition of raw Big Data to Information, Information to Knowledge, and Knowledge to (clinical) Action

There are many alternative models outlining the Big Data infrastructure components and their interrelations. The core of most of them include hardware resources for storing, computing and transferring of information (e.g., data, messages, tools, objects), software library, knowledge base (e.g., expert constructed processing protocols, data, tools and analysis provenance, documentation), and interfaces enabling the human and machine access to the resources and services. Oracle, IBM, Microsoft, Amazon and Google are the established commercial Big Data platforms. Then there are various Federal initiatives including NIH Big Data to Knowledge (http://bd2k.nih.gov) and NSF Big Data (http://www.nsf.gov/cise/news/bigdata.jsp). Finally there is a realm of innovative new open-source projects (e.g., Apache Hadoop [8], MongoDB [9], Node.js [10]) and open-science collaborations (e.g., WEKA [11], tranSMART [12], KNIME [13], Pipeline Workflow [14], Galaxy [15]), among others (http://bd2k.org/BigDataResourceome.html). Fig. 1 illustrates an example of the flow of Big Data analytics, from raw data to information to knowledge and action, using applied clinical neuroscience.

### Existing interactive data visualization platforms

There are a number of recently-developed and powerful data visualization platforms [16]. Many Eyes enables users to upload their multivariate data, generate graphical displays and engage a broader audience [17]. It is a service-based visualization architecture for collaborative creation of rendering of complex datasets that is not open-source. Socrata (www.socrata.com) enables the servicing and sharing of dynamic data from public, government and private organizations. It provides a user-friendly and cost-effective solution for delivering data from sources to destinations (clients) via the Socrata Open Data API (SODA), an open-source interface (http://github.com/socrata). D3 [18] is a modern JavaScript library ubiquitously used for developing dynamic data visualizations. It facilitates effective data import, robust data rendering, and potent variable mappings via a portable browser-mediated graphics. Our restyling tool lets users modify the visual attributes of the marks as well as the mappings from the data to these attributes. Together our tools allow users to easily modify D3 visualizations without examining the underlying code and we show how they can be used to deconstruct and restyle a variety of D3 visualizations. Cytoscape [19, 20] includes a suite of tools for seamless import and export of network and table data, which is available as a stand-alone desktop application and a webapp. Tableau [21] is popular platform for interrogating complex, structured/unstructured, multi-source data. It employs live queries to the data sources to assemble the necessary data and provide multi-faceted data graphics which support on-demand exploratory data analytics. Data Wrangler [22] provides a suite of tools for manipulating and munging incongruent data. Specifically, it allows effective pattern-based data transformations that “clean” the data prior to subsequent data-analytics and visualization.

### Webapp overview

The purpose of the Dashboard project was to design a webapp capable of seamlessly merging datasets from a wide variety of sources without the need for complex mathematical and statistical packages such as R and Mathematica. Two versions of the webapp were designed. The first one (Location-Anchored) provides an interface to fusing examples of available datasets (using a common FIPS or ZIP location mapping) to generate a mashed and munged archive accessible via an intuitive graphical user interface, as well as via a machine accessible API. The second version (Free-Anchored) enables users to provide their own data, map and assemble the data according to specific variables of interest (e.g., subject weight). By using data simulation and by using Crossfilter (http://square.github.com/crossfilter) to dynamically track changes to datasets by using indexing and bitfields, computation time is minimized, thereby providing a fast, scalable, and easy-to-use platform for integrating and comparing datasets from unrelated sources. By integrating a simulation algorithm, the Dashboard maintains data privacy without the need for encryption, making the webapp ideal for consumer or patient based datasets. The use of a non-relational database such as MongoDB allows for built in horizontal scalability, making the app just as proficient at manipulating traditional datasets as it is at true “Big Data” datasets. Data simulation and fusion reduces the overall data footprint of the webapp, resulting in an application that is small enough to deploy onto a smartphone or other portable device. Finally, real-time updating and auto integration of multiple datasets allow for quick and seamless operations without the time overhead associated with traditional RDBMS-style approaches [23]. In order to demonstrate the functionality of the application we have chosen healthcare and neuroimaging data demonstrations. However, the Dashboard webapp can be used to mine, analyze and visualize structured data from virtually any research area.

### Data sources used and rationale for selection

The data sources used to test the Dashboard webapp were selected from a variety of national government and nonprofit online repositories, including the Census Bureau (www.census.gov), Centers for Medicare and Medicaid services (www.cms.hhs.gov), and the Bureau for Labor Statistics (www.bls.gov). These datasets were selected based on completeness, accuracy, and consistent data formatting. Whilst not large enough to serve as true benchmark tests for a Big Data oriented application such as this, the datasets are diverse enough to demonstrate the webapp’s usefulness in cross-discipline applications. For tests regarding properties of the location referenced version of the webapp, data sets that did not contain data for the entire United States were excluded. In addition, data sets that did not display information on a small enough scale were also excluded. For instance, data that only had information on the state level were considered too sparse and were excluded. Particular attention was given to datasets contain information pertaining to the current state of healthcare and medicine in the United States. Furthermore, increased importance was given to datasets containing commonly used demographic identifiers such as race, gender, age group, income, etc. When multiple datasets were encountered containing data for different time periods, sets containing information close to the year 2010 were given priority, in order to match the most recent census year. In order to test the webapp version enabling location-free data references, datasets were chosen that were representative of those typically generated in biomedical research, in this demonstration neuroimaging data. Datasets from multiple studies were used in order to demonstrate the multi-source integration capabilities of the webapp. Listed below is a summary of each included dataset, as well as URLs to each source and rationale for their selection.

2010 Census (http://quickfacts.census.gov/qfd/download/DataSet.txt): Representing the most recent decade-level US census, this dataset provides many of the most commonly used variables for filtering and analysis including race, gender, age group, population density, and education level. This dataset also provides population levels for each county, which are used for a variety of internal dashboard calibrations and operations. This dataset is unique in that it must be included in order for the dashboard to function, and therefore cannot be excluded from the data pool in a custom build of the webapp.

Bureau of Labor Statistics Labor Force Data by County (2010) (http://www.bls.gov/lau/laucnty10.txt): This dataset contains employment statistics provided by the Bureau of Labor Statistics for the year of 2010. This dataset provides several important variables, including unemployment rate and viable labor force levels, and serves as an important indicator of economic health for a particular region.

CMS Hospital Inpatient Data (https://www.cms.gov/Research-Statistics-Data-and-Systems/Statistics-Trends-and-Reports/Medicare-Provider-Charge-Data/Downloads/Inpatient_Data_2011_CSV.zip): Released in June 2014 as part of the Obama administration’s initiative to increase healthcare transparency, this dataset provides various statistics, including average Medicare payment, average withdrawal, and total number of cases. All provided statistics are grouped by inpatient procedure type, as well as by hospital [24].

CMS Hospital Outpatient Data (https://www.cms.gov/Research-Statistics-Data-and-Systems/Statistics-Trends-and-Reports/Medicare-Provider-Charge-Data/Downloads/Outpatient_Data_2011_CSV.zip): Similar to the CMS Hospital Inpatient dataset, this set provides statistics on all outpatient procedures performed by each hospital. Reported statistics are the same as those for the inpatient dataset. This dataset allows for determination of correlations and links between outpatient procedures and socioeconomic factors [25].

2010 Census Economic Survey (ftp://ftp.census.gov/econ2010/CBP_CSV/cbp10co.zip): Provides various economic statistics per county, as well as per industry type, based on North American Industry Classification System (NAICS) specification. Variables reported include number of establishments per industry type, average payroll per industry type, and number of establishments per industry type per size class.

CMS Physician Data (http://download.cms.gov/Research-Statistics-Data-and-Systems/Statistics-Trends-and-Reports/Medicare-Provider-Charge-Data/Downloads/Medicare-Physician-and-Other-Supplier-PUF-CY2012.zip?agree=yes&next=Accept): Released April 2014 as part of the White House’s initiative to improve healthcare transparency, this report gives provides information and statistics on individual practitioners, as well as group practices. Statistics reported include provider credentials, provider gender, specialization, average Medicare withdrawal, and patient count. In addition, specific information is given for each type of condition treated.

Biomedical Data: We used clinical, imaging and demographic data from a Parkinson’s disease study [26, 27], a nutrition and obesity study [28, 29], and an Alzheimer’s study [30, 31]. These datasets were chosen to test and validate the performance of the Dashboard app for several reasons. First, these datasets represent large, heterogeneous, multi-source, incomplete and longitudinal Big Data. Second, the translational research projects managing the data provided powerful driving biomedical challenges of interrogating and mining the complex data to visually identify patterns trends and associations. And third, they did not explicitly include FIPS/ZIP code meta-data to allow seamless integration with the default Dashboard datasets, which provided the opportunity to validate the webapp interface with non-standard data and establish indirect mapping anchoring cases by alternative meta-data (e.g., subject weight).

### Data conversion and quality control

**Fig. 2:**
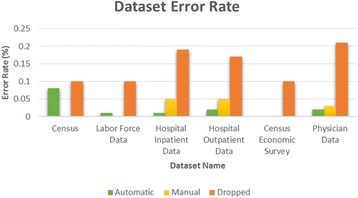
Graph of rate of automatic fixes, manual fixes, and data point drops for each data set

Data conversion first began by ensuring that all datasets were stored under a common .csv file type. Any datasets that used alternate file types (.xls, .json, .pdf, etc.) were first converted to ASCII csv files using a standard conversion utility. Next, all files were preprocessed in order to remove invalid data points. In addition, data points with incorrect identifying information were attempted to be fixed, both via automatic C++ scripts, as well as through manual inspection. Any remaining incorrect data points that were unable to be rectified were then discarded. Additionally, data points were inspected to ensure a low error rate. Fig. 2 demonstrates the proportions of encountered errors that were either fixed (automatically or manually) or dropped. Overall, the error rate for all data points was maintained at less than 0.25 %. After the data-scrubbing and preprocessing, the datasets were converted into a form suitable for database processing, operations and fusion. A custom script was designed for each dataset which converted all data into key-value pairs representing discrete data points for each county code. In addition, the script encoded relevant information for each variable, including the variable’s name, source, and data type. The processed data file was then stored in JSON format in order to facilitate the operations performed in the second stage.

### Data fusion

Data representation and manipulation was done via simulation of a 1/1,000^th^ population sample. Qualitative properties (Race, Gender, etc.) were assigned via simple random sample, using probability distributions calculated from county-level data variables. Quantitative variables (income, unemployment rate, etc.) were set equal to state or county averages, with no distribution or simulated deviation from the mean. By simulating data instead of performing direct filtering and comparison operations, correlations and statistical operations can be performed without revealing any information about the true raw data points. This allows for a layer of anonymity between data and statistics, thereby allowing for increased privacy in applications involving personal or confidential information. Furthermore, the simulation layer results in a fused dataset much smaller in size than the sum of each of its corresponding raw files, thereby enhancing the portability of the webapp.

### Service infrastructure

Once all raw data had been processed, a separate database handler program, Node JavaScript, exported all processed data into a non-relational database [10]. A significant point to note is that the same database handler program was used for all datasets and variables, in contrast to the previous step, which required a separate conversion protocol for each dataset in order to accommodate the variety of file types and data formats. The webapp uses MongoDB as the non-relational data server [32]. In addition to exporting data, the database handler also generates a summary file containing metadata for each of the datasets, as well as their respective variable sets. This meta-information was made available to end users via an API interface. In addition, a second API interface was established to allow machine access to the final simulated data points.

As an alternative to the machine-compatible web API’s, a GUI based dashboard webapp was created to facilitate human interaction with the integrated dataset. The dashboard graphical interface operates by creating and manipulating charts to observe the relationships between datasets. Chart creation and manipulation options are performed via an intuitive drag-and-drop tile based system. Graphs are generated through interaction with a creation wizard, allowing the end-user to specify data-sources, data-variables, chart type, and a variety of other customization features. Options and available chart types are context-sensitive, and will change based on properties of the selected data set (i.e., qualitative datasets will have a different selection of available chart types than discrete quantitative data sets). Available chart types include commonly used graphing utilities [33–35]:

- Pie Chart
- Doughnut Chart
- Bar Graph
- Histogram
- Geo Choropleth Chart
- Bubble Chart
- Scatterplot
- Box Plot

In addition, several “widgets”, such as a table viewer, are provided in order to further enhance the effectiveness and usability of the webapp.

Once generated, charts and other widgets can be freely moved around the dashboard in accordance with the end-user’s needs. In addition, selection a certain value or range of values causes the population to be filtered to only display samples with the selected value (s). Since all charts and widgets share the same population pool, any filters or operations applied to a chart are reflected on all other charts. For instance, selecting the *male* category in a *Gender* pie chart will cause all other charts to filter data so that only male samples are included. This demonstrates the visual graphic based, rather than SQL based, query of the mashed data archive.

Loading the dashboard webapp in the web-browser instantiates the downloading of the summary file from the MongoDB webserver. This allows the webapp to populate the list of all available variables which can be used by the user to initiate the graphical query process. Upon selection and creation of a chart, the webapp queries and downloads the specific data for the required variable (s), and integrates the data into the simulated population dataset. The simulated population pool consists of approximately 300,000 individual “samples”, with each sample representing 1,000 individuals. In order to integrate a new variable, a probability distribution is generated for each possible value, and each sample is assigned a value using a simple random sample based on the calculated probability distribution (e.g. a county with 3,000 males and 5,000 females would be represented as 8 samples, of which on average 3 will be male, and 5 will be female).

The dashboard webapp also allows for the ability to aggregate multiple variables using simple operations. By selecting the “Customize” tile, users are able to add, multiply, or subtract variables, allowing for creation of more complex combined variables.

The current implementation of the dashboard webapp allows for an integration and manipulation of a maximum of 30 different charts at a time, where each chart can contains between 1 and 6 variables. However, we have observed speed and performance impacts as the number of charts doubles (e.g., from 8 to 16 charts).

In addition, the webapp was designed to be able to accommodate a near-limitless number of datasets and variables without suffering any significant performance issues. Currently, approximately 700 different variables are available, though more are expected to be added in the near future as we expand the integrated archive to include complementary information. Finally, we plan to allow for mashing of user-submitted datasets, in order to further enhance the versatility, usability and customization of the webapp.

### Data retrieval

Filtered and modified data can be exported from the application in a variety of ways, in order to allow further data analysis via other programs and applications. The full combined data file, as well as all source code for the webapp can be found at the project’s GitHub page at https://github.com/SOCRedu/Dashboard-Lab. Processed datasets can be downloaded in zip format at http://socr.umich.edu/data/SOCR_DataDahboard_Dataset_V1.1.zip.

In order to facilitate machine data retrieval, two data API’s have been provided. A raw data REST API, located at http://socrdev.nursing.umich.edu:8080/users/var/[VARNAME] allows for retrieval of unfiltered quantitative variable data points. Qualitative variable data points can similarly be retrieved from the REST API at http://socr-dev.nursing.umich.edu:8080/users/super/[VARNAME].

A second data API, located at http://socr-dev.nursing.umich.edu:8080/api/request, was implemented in order to allow for customized data filtration procedures. By utilizing a Node based server-style version of CrossFilter [36], the API allows for data integration and filtration with results identical to the GUI Dashboard webapp. The webapp utilizes a query format for requests, wherein filtered variables are specified by setting the variable to the requested filtered variable, and requested variables are indicated by specifying the value of the reqVar variable. For example, in order to request data points for the Average Medicare Payment for Extracranial Procedures for patients with income greater than 60000 and are either White or Black, the API would be queried as http://socr-dev.nursing.umich.edu:8080/api/request?income_per_capita[min]=60000&race=White&race=Black&reqVar=EXTRACRANIAL_PROCEDURES_W-O_CC-mcc_avg_charge.

Besides the machine accessible REST API’s, data from the dashboard can also be exported using the Dashboard web application itself. At any time during operation of the webapp, the Export tile (button) can be selected in order to download all data to local storage. Only data points that pass all currently applied filters are exported, and only variables which are currently displayed on the Dashboard are represented in the exported data file. For example, clicking the Export tile after selecting ‘Male’ on a Gender pie chart and selecting income ranges from $40,000 to $60,000 on an income histogram will generate a CSV data file containing income levels and gender for all Males with income between $40,000 and $60,000.

### User-submitted datasets

**Fig. 3:**
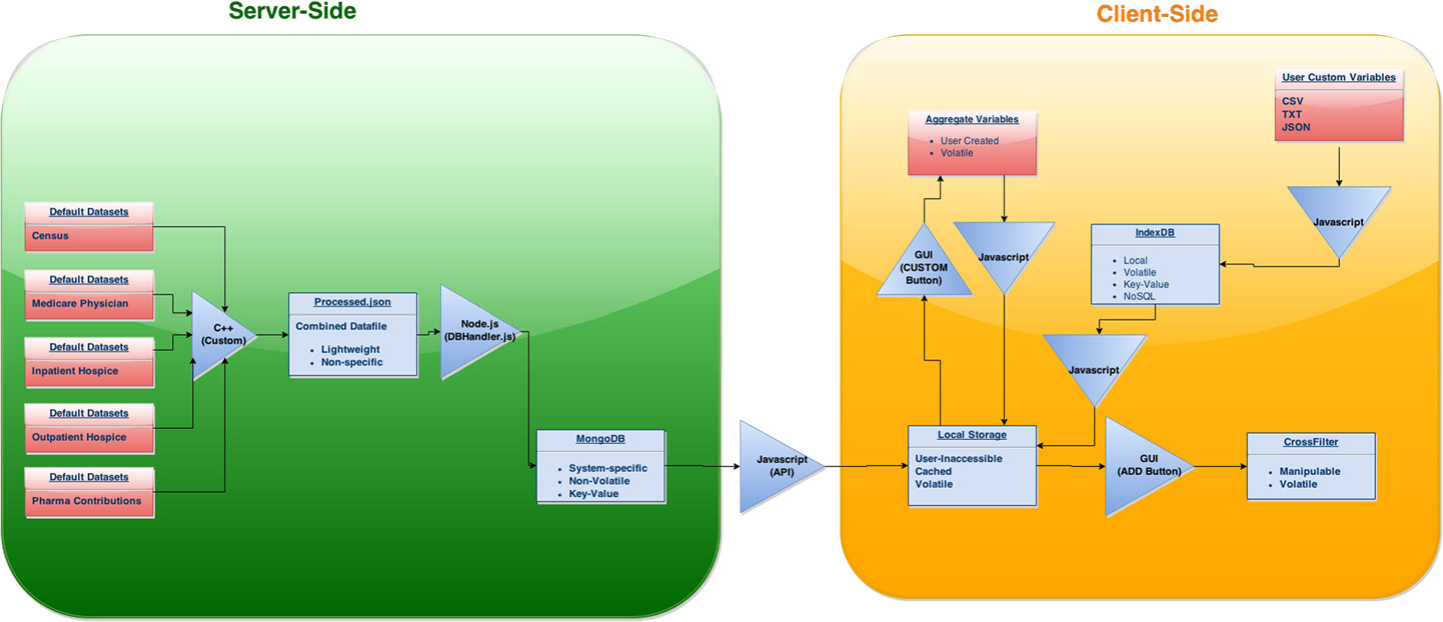
Dashboard dataflow – note the unidirectional flow from server side (green) to client-side (orange)

A data import utility was created in order to allow uploading of custom datasets by the end-user without the need for any knowledge of programming and data modelling. User datasets may be loaded as .csv files using the “Import” option in the Dashboard. These data are stored client-side using IndexedDB, a browser-implemented non-relational database. Entries in the dataset are then extracted, and grouped into cohorts based on the variable and range designated by the end-user. Variables are automatically designated as quantitative or qualitative based on entries, without the need for any user-provided metadata information. The converted data is then passed into Crossfilter, where it can then be used in conjunction with other user-provided datasets, as well as preexisting datasets downloaded from the server. Fig. 3 demonstrates the overall dataflow for the webapp.

### Data interrogation examples

Below we include three example use-cases for the dashboard webapp, which highlight the major features of the application, as well as its practicality in performing cross-dataset comparisons. The webapp can be accessed online at http://socr.umich.edu/HTML5/Dashboard/.

**Fig. 4:**
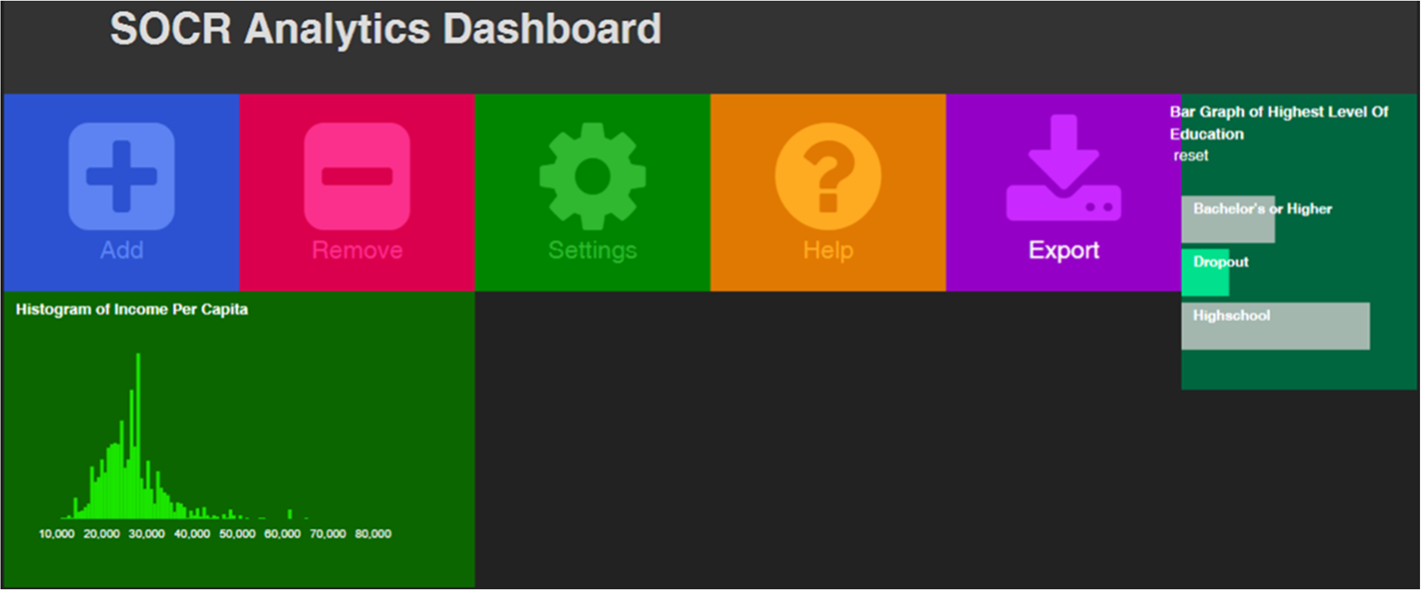
Resulting chart after performing the steps outlined in the first use-case illustrating the generation of a pair of charts

**Fig. 5:**
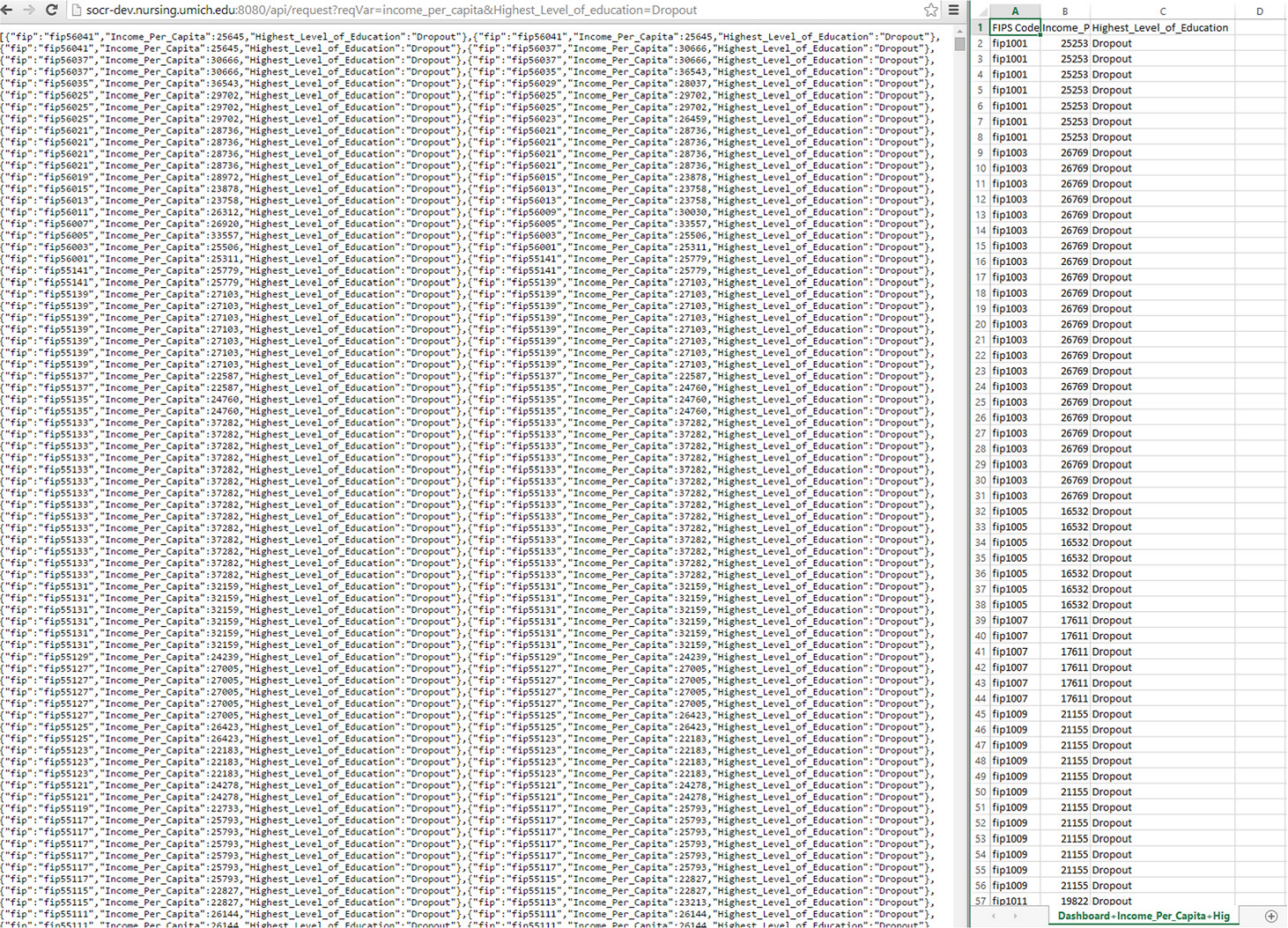
Parallel between the machine API interface (left) and human data export GUI interface (right) and for query and retrieval of information from the data Dashboard

The first example gives a general overview of the chart creation process, as well as a depiction of the usefulness of the dynamic global filtration feature, Fig. 4:

- Upon startup of the webapp, select the ‘Add’ tile.
- In the ‘Data Source’ dropdown, select 2010 Census
- In the Data Variable dropdown, select Highest Level of Education
- Press the Continue Button
- In the chart selection window, select the bar chart.
- Press the Continue button
- Once again, select the ‘Add’ tile
- In the ‘Data Source’ dropdown, select 2010 Census
- In the Data Variable dropdown, select Income per Capita
- Press the Continue Button
- In the chart selection window, select the histogram
- Press the Continue button
- It is now possible to observe relations between the two datasets by applying filters on one or both of the charts. For example, select the ‘Dropout’ bar on the Highest Level of Education bar chart. There should be an observable decrease in the Income per Capita histogram, indicating a correlation between level of education and income. Pressing the export button will download the resulting dataset as a csv file. The resulting file is the CSV equivalent of the result from an API call to http://socrdev.nursing.umich.edu:8080/api/request?reqVar=income_per_capita&Highest_Level_of_education=Dropout, Fig. 5.

**Fig. 6:**
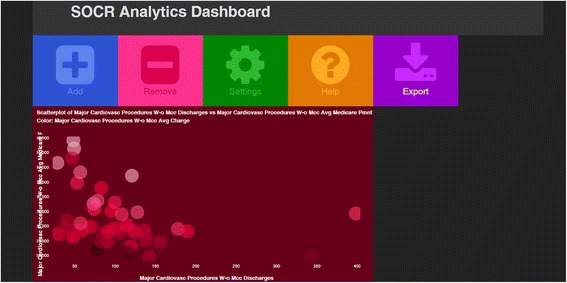
Scatterplot showing negative correlation between the number of cardiovascular procedures performed and the average cost per procedure

**Fig. 7:**
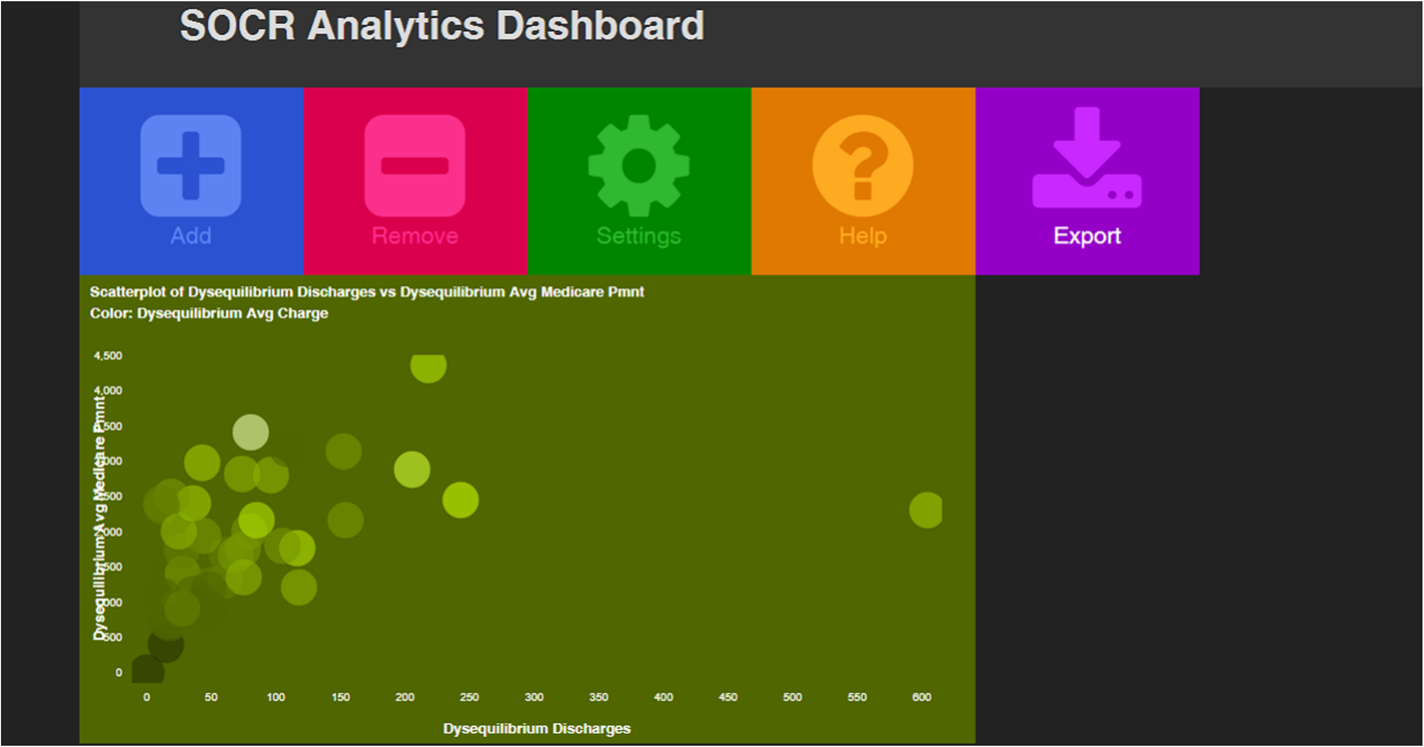
Dashboard scatterplot showing positive correlation between the number of disequilibrium procedures performed and the average cost per procedure

The second example describes usage of multi-dimensional charts and illustrates the usefulness of the webapp in drawing novel conclusions related to socio-economics and health care, Fig. 6 and Fig. 7:

- Select the Add tile
- In the ‘Data Source’ dropdown, select Inpatient Charge Data (Total discharges)
- In the Data Variable dropdown, select Major Cardiovascular Procedures (Total Discharges)
- Press the Continue Button
- In the chart selection window, select the scatterplot
- Press the Continue button
- In the ‘Data Source’ dropdown for the Y axis, select Inpatient Charge Data (Avg medicare pmnt)
- In the Data Variable dropdown for the Y axis, select Major Cardiovascular Procedures (Avg medicare pmnt)
- In the ‘Data Source’ dropdown for Color, select Inpatient Charge Data (Avg covered charge)
- In the Data Variable dropdown for Color, select Major Cardiovascular Procedures (Avg covered charge)
- Press Continue
- In the resulting scatterplot, there is a fairly strong observable *negative* correlation between number of discharges and per-patient charge for hospital treatments of Major Cardiovascular Procedures. However, if the above steps are repeated for Disequilibrium, there is instead a *positive* correlation.

**Fig. 8:**
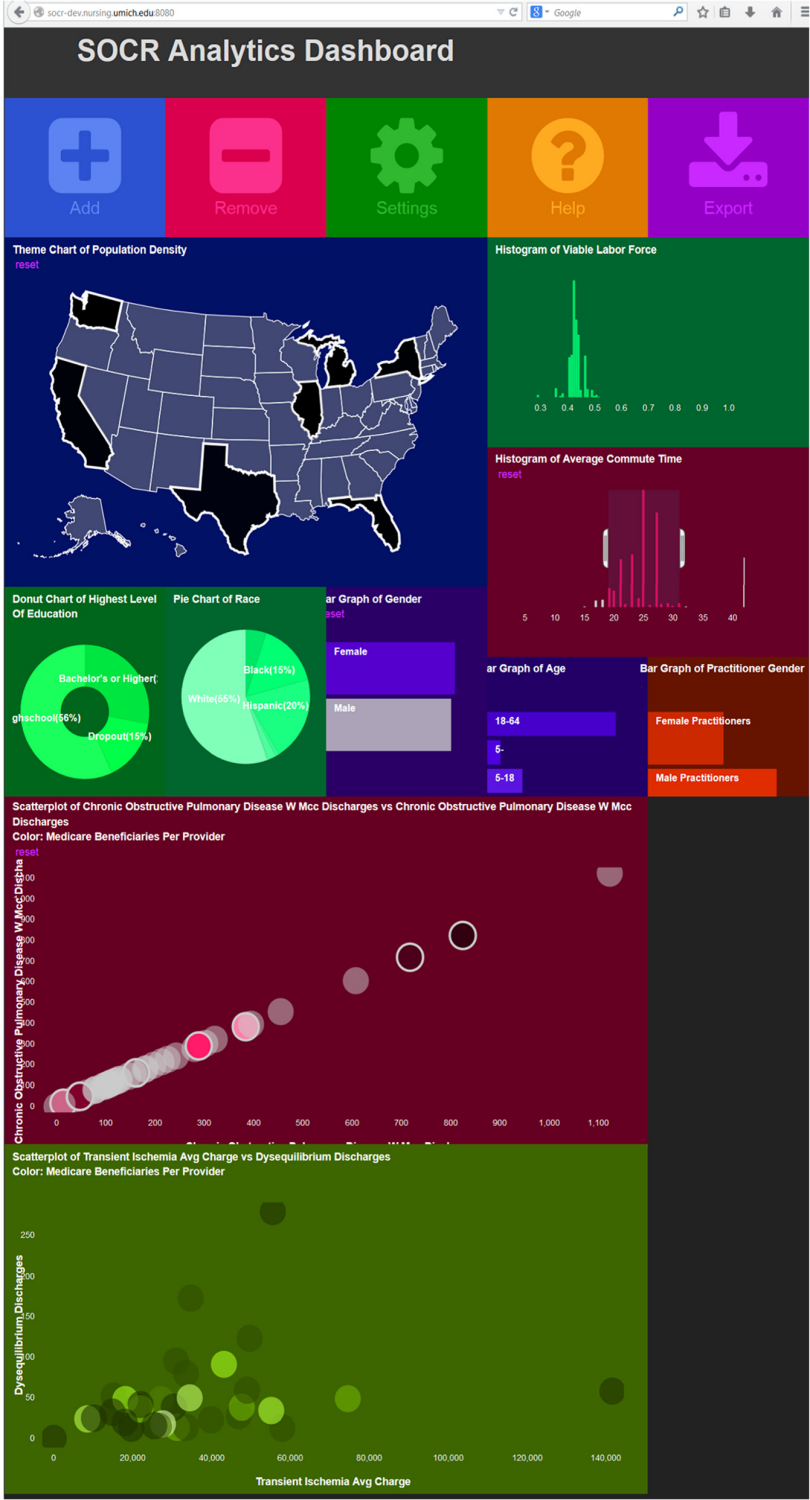
Sample of a dashboard instance investigating the associations between a dozen variables from all sources. User selections of cohorts stratifying the data on any of these data elements propagate to impact all other displays. For instance, narrowing the gender to females only will cause a smooth dynamic morphing of all charts limiting to females the data for each displayed variable. In this example, the highlighted states (WA, CA, TX, FL, IL, MI, NY) in the US geographic map, the vertical limit bars on the histogram of the Commute Times, and the highlighted blobs in the Chronic Obstructive Pulmonary Disease scatter plot/bubble chart all indicate stratification parameters narrowing the data search to the specified cohort demographics and phenotypic traits

Figure 8 shows a collage of 12 heterogeneous plots representing the graphical query using the Dashboard human GUI interface. It shows a wide variety of univariate, bivariate and geographic charts available in the webapp. This example is interesting as it demonstrates the integrated nature of the mashed dataset and the core functionality of the Dashboard to enable traversal, navigation, search and exploration of multivariate associations in large and diverse sets of information.

The third example demonstrates the effectiveness of the Dashboard for multi-source neuroimaging data analysis. The example incorporates data points from both the Parkinson’s and Alzheimer’s disease studies and groups data points into cohorts based on their cerebellum volume, which is mostly unaffected in neurodegenerative disorders.

- Select Free-Anchored Version
- For the Cohort Name, enter select *cerebellum_Volume*
- Ensure that both datasets contain a column with a header named *cerebellum_Volume*
- Select the cohort range to be from 0 to 300000
- Select number of cohorts to be 300
- Import both datasets by selecting the ‘Import’ tile and navigating to the dataset .csv files

**Fig. 9:**
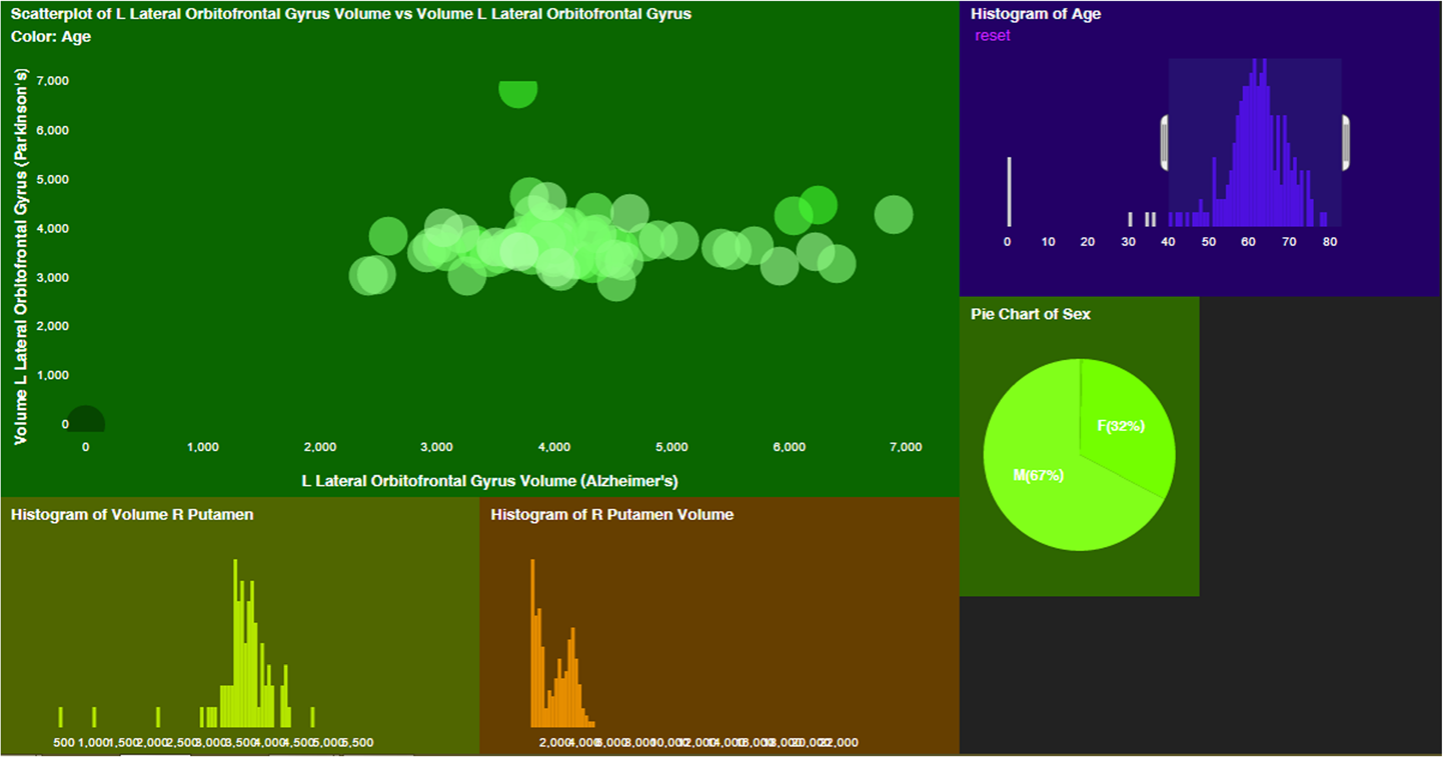
Sample comparisons created using Alzheimer’s and Parkinson’s data, as outlined in the third example. Note the filter on the age histogram, in order to select patients with ages greater than 40

As can be seen in the above example, importing and integrating user-supplied datasets is fairly streamlined, with very little preprocessing required. Fig. 9 shows several resulting graphs created from the above method, including a scatterplot comparing orbitofrontal gyrus volumes for both Alzheimer’s and Parkinson’s patients.

### Performance analysis

The data upload and conversion feature of the dashboard was analyzed to gauge dashboard performance for various datasets. Testing was done Using Chrome v.42.0.2311.152 m browser running on a Windows 8.1 Operating System, Intel Core i5 processor, 8 GB of RAM, 320 GB SATA hard drive, and 100 MB network connection. Randomly generated datasets were created using a predetermined number of variables and entries. Dataset upload and graph generation times were obtained using the Google Analytics User Timing library (analytics.js).

Table 1 demonstrates data upload and graph generation times for datasets containing 10 variables with varying number of entries. 10 datasets were generated for each entry-length category. As can be seen, whereas upload time increased relatively linearly with increasing size, graph generation time experienced little to no change. This demonstrates the effectiveness of the simulation method in reducing graphing and filtering runtimes for extremely large datasets.

**Table 1:** Data upload and graph generation times based on number of entries per variable

Number of entries	Mean upload time (ms) (*n* = 10)	Std deviation (ms)	Mean graph generation time (ms) (*n* = 10)	Std deviation (ms)
100	128	37	952	244
1,000	165	29	1,215	216
100,000	14,225	1,564	1,093	169

Table 2 demonstrates data upload and graph generation times based on the number of variables per dataset. Datasets were generated with 1,000 entries per variable, with the number of variables ranging from 100 to 10,000. As the results show, upload time increased with increasing number of variables, whereas graph generation time remained relatively constant. This demonstrates the efficacy of the Dashboard in generating graphs and applying filters regardless of the number of variables present

**Table 2:** Data upload and graph generation times based on number of variables

Number of variables	Mean upload time (ms) (*n* = 10)	Std deviation (ms)	Mean graph generation time (ms) (*n* = 10)	Std deviation (ms)
100	992	62	1,433	367
1,000	2,265	456	1,978	725
10,000	98,395	8,289	919	126

## Discussion

The era of Big Data deluge presents unique challenges and opportunities for innovation to enhance, expedite and simplify the processes of data exploration, query and discovery. Interactive and device-agnostic tools and services facilitating such data munging and interrogation are necessary for the next generation of powerful analytics-driven modeling, prediction and decision-making. In this manuscript we report on the construction of an interesting mash of multi-source data that could be used to explore intricate associations between variables, population strata, or clusters of data elements, which may not be easy to untangle by independent inspection of the individual data archives. In addition, we present a new platform and device agnostic tool (Dashboard webapp) for querying, navigating and exploring the multivariate associations in complex heterogeneous datasets.

We chose to illustrate the core functionality and service-oriented infrastructure supporting this application using healthcare data. Specifically, the current version of the webapp used US data from the 2010 Census (Demographic and Economic surveys), Bureau of Labor Statistics, and Center for Medicare Services (Hospital Inpatient and Outpatient, Physician Data). This dashboard platform is continuously expanded to include additional data elements and can be customized to manage diverse types of datasets with varying applications. This framework could be used for exploratory analytics, confirmatory analyses, meta-analyses, as well as for education and training purposes.

This entire framework is developed under open-science principles and facilitates the collaboration, refactoring, improvement and sustainability of the data, software tools, web-services and computational infrastructure developed to harvest, pre-process, munge, fuse, query, analyze and interrogate the integrated archive. The complete dataset is available online (http://socr.umich.edu/data/SOCR_DataDahboard_Dataset_V1.1.zip), the webapp can be openly accessed on a public server (http://socr.umich.edu/HTML5/Dashboard/) and the complete software infrastructure is on GitHub (https://github.com/SOCRedu/Dashboard-Lab and https://github.com/SOCR).

## Conclusions

In this manuscript we report on the design, implementation and testing of a new platform, SOCR Data Dashboard, for exploratory querying of heterogeneous and multi-source datasets. The Dashboard architecture enabled graphical navigation and discovery of subtle associations between data elements, sub-population strata, or clusters that may be obfuscated during traditional protocols for data inspection. The platform is open-source and openly disseminated as source-code and as service-oriented infrastructure. We tested the Dashboard using complex data from the 2010 US Census, Bureau of Labor Statistics, Center for Medicare Services, and various neuroimaging studies of neurodegeneration. We use continuous-development and extreme-programming practices to rapidly design, implement, test, update, and distribute the data archive and the dashboard human and machine interfaces. The entire computational and data science community is encouraged to employ, extend and support the Dashboard platform for research and training in exploratory analytics, confirmatory analyses and meta-analyses.

## Appendix

### Data dashboard API schema

The REST APIs provides a way to read data from SOCR resources. Every request should include with this prefix “http://socr-dev.nursing.umich.edu:8080/api/request?” followed by the parameters and values in the URL. The operator “&” is used to separate the different parameters and the operator “=” is used to set the value for a particular parameter. The requested parameter should be stated as “reqVar”. For quantitative parameters, input the parameters followed by “[min]” and/or “[max]” to indicate the desired range. For example, if the requested range for income is between 40000 and 80000, then the input should be “income [min] = 40000&income [max] = 80000”. If only the “[min]” is stated, then the “[max]” is assumed to be the highest number in that particular parameter. If only the “[max]” is stated, then the “[min]” is assumed to be the lowest number in that particular parameter.

### Best practices

- Make sure all parameters are properly URL encoded.
- Limit requests to 10 parameters.
- Requests should not be too complex.

### Example searches

- You want the unemployment rate for White or Black male, your search URL would be:
- http://socrdev.nursing.umich.edu:8080/api/request?gender=Male&race=White&race=Black&reqVar=unemployment_Rate
- You want the Average Medicare Payment for Extracranial Procedures for patients who are 65 and older, your search URL would be:
- http://socrdev.nursing.umich.edu:8080/api/request?age=65%2b&reqVar=EXTRACRANIAL_PROCEDURES_W-O_CCMCC_avg_medicare_pmnt
- If you want the Average Medicare Payment for Extracranial Procedures for patients who are 65 and older and are White or Black, your search URL would be:
- http://socrdev.nursing.umich.edu:8080/api/request?age=65%2b&race=White&race=Black&reqVar=EXTRACRANIAL_PROCEDURES_W-O_CC-MCC_avg_medicare_pmnt
- If you want the Average Medicare Payment for Extracranial Procedures for patients who are White or Black and have income above 60000, your search URL would be:
- http://socrdev.nursing.umich.edu:8080/api/request?age=65%2b&race=White&race=Black&reqVar=EXTRACRANIAL_PROCEDURES_W-O_CC-mcc_avg_charge
